# Case Report: Cervical internal vertebral venous plexus thrombosis diagnosed using time-of-flight magnetic resonance angiography in a dog

**DOI:** 10.3389/fvets.2026.1778813

**Published:** 2026-03-11

**Authors:** Rute Canejo-Teixeira, Aurelia Gasser, Daniela Schweizer, Christina Precht

**Affiliations:** 1 Division of Clinical Radiology, Department of Clinical Veterinary Science, Vetsuisse Faculty, University of Bern, Bern, Switzerland; 2 Division of Clinical Neurology, Department of Clinical Veterinary Science, Vetsuisse Faculty, University of Bern, Bern, Switzerland

**Keywords:** cervical spine, dog, internal vertebral venous plexus, thrombosis, time-of-flight magnetic resonance angiography

## Abstract

**Background:**

Time-of-Flight (TOF) magnetic resonance angiography (MRA) is a non-contrast imaging technique used for vascular assessment but underexplored in veterinary spinal imaging. This case report presents the use of TOF-MRA at 3 Tesla to diagnose cervical internal vertebral venous plexus (IVVP) thrombosis in a dog, advancing the application of three-dimensional imaging in veterinary neurology.

**Case presentation:**

A 13-year-old castrated male Siberian Husky presented with acute cervical pain, left thoracic limb lameness, and generalized tonic–clonic epileptic seizures. Laboratory findings revealed mild neutrophilia (10.83 × 10^9/L), elevated fibrinogen (404 mg/dL), and a urinary tract infection (bacteriuria, leukocyturia, and proteinuria).

**Diagnosis and outcome:**

Pre- and post-contrast MRI examination of the head revealed no abnormalities of the brain. A delayed post-contrast conventional T2-weighted MRI sequence of the cervical spine demonstrated focal loss of normal flow void of the left IVVP at the C4-C5 level. Three-dimensional TOF-MRA showed a corresponding focal signal void in the left ventral IVVP from C4 to C5 and an associated venous dilation consistent with thrombosis.

**Conclusion:**

This case demonstrates TOF-MRA’s potential as a non-invasive, contrast-free method for diagnosing spinal venous thrombosis in dogs. It underscores the importance of vascular imaging in dogs with acute neurological signs, offering valuable insights for veterinary practice and future research.

## 1 Introduction

Time-of-Flight (TOF) magnetic resonance angiography (MRA) is a non-contrast technique that exploits flow-related enhancement to depict vascular structures with high signal intensity against a suppressed background of stationary tissues (1, 2). In TOF sequences, repeated radiofrequency pulses saturate static parenchyma within the imaging slice—causing stationary spins to loose longitudinal magnetization and appear dark—whereas unsaturated spins present in the inflowing blood retain high signal, rendering vessels bright and eliminating the need for gadolinium-based contrast medium (1–3).

Three-dimensional (3D) TOF scans, particularly at higher field strengths such as 3 Tesla (T), allow thin-slice acquisitions with submillimeter spatial resolution and multiple multiplanar reformations, enabling detailed visualization of small vessels and collateral networks (1, 4). In human medicine, TOF-MRA has been used in neuroimaging for the non-invasive assessment of intracranial aneurysms (18), for detection of intracranial atherosclerotic steno-occlusive disease (19) and has been shown to be effective in the detection of cerebral venous sinus thrombosis (20). Findings such as flow voids, focal outpouchings and signal changes may parallel the thrombosis and venous enlargement observed in canine patients, suggesting TOF-MRA’s potential utility in veterinary spinal vascular assessment.

In veterinary medicine, however, TOF-MRA’s application remains limited. At 1.5 T, it has been used to evaluate intracranial arteries in dogs (5), reliably delineating major vessels like the middle cerebral and basilar arteries; however, visualization of smaller branches (e.g., rostral cerebellar arteries) was inconsistent (2). Contrast-enhanced 3D CTA has mapped the canine lumbar arterial supply and internal vertebral venous plexus (IVVP) in healthy dogs (6, 7), but TOF-MRA’s ability to visualize pathological venous lesions without gadolinium remains underexplored in veterinary spinal imaging.

Previous reports of vertebral venous thrombosis in dogs have relied on conventional MRI sequences, often with gadolinium enhancement, to infer thrombus presence via heterogeneous signal or secondary venous enlargement (8). In contrast, TOF-MRA can directly depict intraluminal flow interruption as a signal void, distinguishing it from slow flow or hypoplastic vessels (9). The use of TOF MRA at 1.5 T his has recently been shown to detect a persistent thrombus in the IVVP of an adult dog (10), thus confirming the usefulness of the technique in canine patients.

This case report describes a 13-year-old castrated male Siberian Husky imaged at 3 T for acute cervical pain and neurologic signs. A 3D TOF sequence revealed a focal signal void within the left ventral branch of the IVVP extending from C4 to C5 consistent with thrombosis and associated venous enlargement, corresponding to an area of loss of normal flow that had been considered suspicious on routine T2-weighted sequences. This case illustrates the value of TOF-MRA at 3 Tesla to identify a cervical IVVP thrombosis in a dog, highlighting its potential as a non-invasive diagnostic tool in veterinary neurology.

## 2 Case description

A 13-year-old male castrated Siberian Husky (weighing 18.2 kg) was referred to the Neurology Department of the Small Animal Hospital at the Vetsuisse Faculty, University of Bern, with a history of a single yelp during a morning walk, ambulatory cervical pain when handled, limping in the left thoracic limb and two generalized tonic–clonic epileptic seizures with involuntary urinary incontinence. The patient had a history of an untreated perineal hernia, unspecified gastrointestinal issues managed with diet and was receiving routine preventive management for obstipation.

### 2.1 Clinical investigation

During general clinical examination, the dog exhibited marked stress but showed no significant abnormalities on physical assessment; however, he voided odorous urine. During the neurological examination, the dog was alert but continued to exhibit previously noted stress-related behavior. The initially observed cautious gait with left thoracic limb lameness was no longer apparent; however, marked pain turning the neck to the left remained. The remainder of the neurological examination was unremarkable. As no neurological defects other than cervical pain were present on examination, spinal cord involvement could not be confirmed, and the neuroanatomical localization was therefore considered to be the cervical region without evidence of spinal cord involvement. Owing to the history of generalized tonic–clonic seizure activity, a forebrain localization could not be excluded based on the anamnesis alone despite the absence of abnormalities on neurological examination.

Initial blood work was unremarkable, and the patient was hospitalized overnight for pain management. Overnight, the patient developed generalized tonic–clonic seizures with loss of consciousness. Repeat blood work the next day revealed mild neutrophilia (10.83 × 10^9 cells/L; reference 2.6–8.9 × 10^9 cells/L), elevated fibrinogen (404 mg/dL; reference 109–311 mg/dL) with normal PT (7.3 s; reference 5.7–8.5 s), PTT (11.6 s, reference 9.6–14.3 s) and albumin (33 g/L; reference 22–39 g/L). Urinalysis showed bacteriuria (+++), leukocyturia (20–30 cells), and proteinuria (25 mg/dL). Because of severe cervical pain, seizure activity, the positive bacterial urine culture, as well as the occurrence of a generalized seizure, MRI of the brain and cervical spine was performed. Considering the patients age, thoracic radiographs and abdominal ultrasound were recommended prior to MRI to screen for metastatic disease.

### 2.2 Imaging diagnosis

Laterolateral thoracic radiographs in left and right lateral recumbency revealed the presence of multiple small round and well-defined mineral-opaque structures of less than 2 mm diameter within the lung field suspected to be pulmonary osteomas, but were otherwise unremarkable. Abdominal ultrasonography showed mild homogenous hepatopathy, mild cholecystolithiasis and incidental right renal cyst. Brain MRI at 3 T (Siemens MAGNETOM Vida, Siemens Healthineers International AG, Zürich, Switzerland) was performed with the routine set of sequences including pre- and post-contrast T1 weighted (w), T2w, T2-FLAIR, T2*w and diffusion weighted sequences. This was followed by a MRI examination of the cervical spine 45 min after the administration of contrast medium (Acidum gadotericum 0.5 mmoL/mL at a dosage of 0.3 mL/kg body weight; Clariscan, GE Healthcare AG, Opfikon, Switzerland) including following sequences: T2w Sampling Perfection with Application optimized Contrasts using different flip-angle Evolutions (T2-SPACE) (TE = 223 ms, TR = 1970 ms, FA = 100, slice thickness = 2 mm, no gap) in sagittal plane; T2w turbo spin echo (TSE) in sagittal (TE = 84 ms, TR = 2,800 ms, FA = 120, slice thickness = 2.5 mm, gap = 0.33 mm) and transverse plane (TE = 90 ms, TR = 4,950 ms, FA = 120, slice thickness = 3 mm, gap = 0.75 mm); T1w TSE in transverse plane (TE = 9.4 ms, TR = 3,000 ms, FA = 159, slice thickness = 3 mm, gap = 0.75 mm); T2*w gradient echo (GE) fast low-angle shot echo-planar–based (FLED) in transverse plane optimized for hemorrhage detection (TE = 7.38 ms, TR = 574 ms, FA = 20, slice thickness = 3 mm, gap = 0.75 mm); short tau inversion recovery (STIR) in dorsal plane (TE = 38 ms, TR = 4,000 ms, TI = 220 ms, FA = 130, slice thickness = 3 mm, gap = 0.6 mm) and a 3D TOF (TE = 3.42 ms, TR = 21 ms, FA = 19, slice thickness = 0.5 mm, no gap). The patient was premedicated (acepromazine 0.005 mg/kg) and anesthesia was induced with midazolam (0.2 mg/kg) and propofol (1 mg/kg). The patient was intubated with a 12 mm endotracheal tube, placed in sternal recumbency for the brain MRI and dorsal recumbency for the cervical spine MRI and monitored throughout the study while anesthesia was maintained with isoflurane (with a FiO_2_ of 65–85%).

#### 2.2.1 Minor findings

On brain imaging, the left tympanic bulla was filled with T2w, T2w FLAIR, and T1w hyperintense and non-contrast enhancing material. The left tympanic bulla wall was well delineated, with contrast enhancement of its luminal lining and of the external ear canal lining. The inner ear and adjacent soft tissues were unremarkable. These findings were considered consistent with left sided otitis media and externa without involvement of the inner ear.

On cervical MRI a mild, heterogeneous T2w hypointensity of the nuclei pulposi of the intervertebral discs of the cervical spine was noted. Narrowing of the intervertebral disc space at C5/C6 was present, with multifocal mild intervertebral disc protrusions at C2/C3, C3/C4, C4/C5, and C5/C6, resulting in a focal thinning of the ventral subarachnoid space at C3/C4. No narrowing of the neuroforamen was noted. At the cranio-dorsal aspect of C7, a T2w hyperintense area was noted that suppressed on STIR. Findings were considered consistent with multifocal intervertebral disc degenerations without spinal cord compression and focal fatty conversion of bone marrow at C7.

#### 2.2.2 Major findings

**Figure 1:**
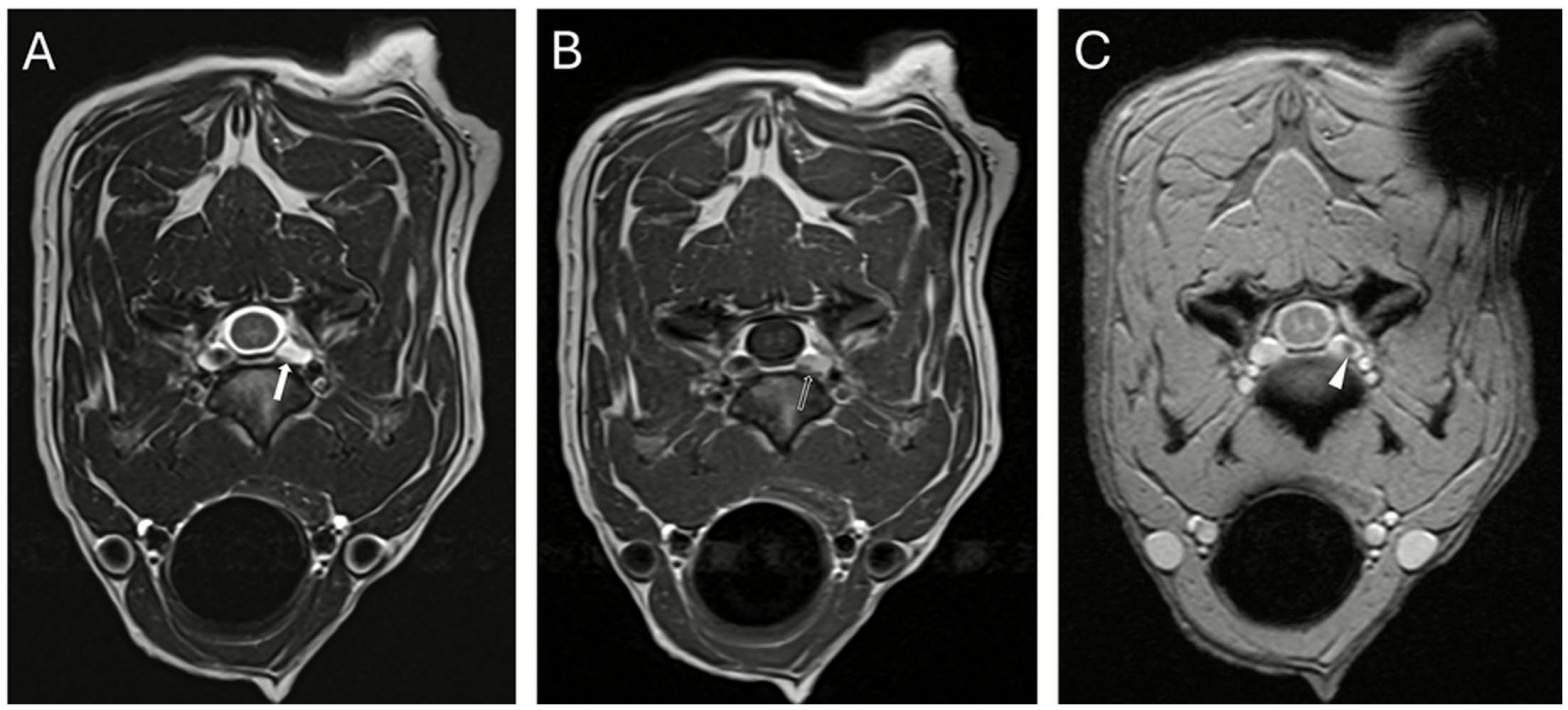
Transverse T2w TSE **(A)**, delayed post-contrast T1w TSE **(B)**, and T2*w GE FLED **(C)** sequences at the level of C4-C5. A focal loss of the normal flow void within the left internal vertebral venous plexus is present on the T2w sequence (**A**, white arrow). Corresponding focal hyperintense signal on the T1w sequence (**B**, open white arrow) and susceptibility artifact on the T2*w GE FLED optimized for hemorrhage detection (**C**, arrowhead).

The transverse T2w sequence on cervical MRI, demonstrated focal loss of the normal flow void of the left IVVP at the C4-C5 level, a corresponding focal hyperintense signal on the T1w sequence and a susceptibility artifact on the T2*w GE FLED optimized for hemorrhage detection (Figure 1). Because the cervical spine MRI was performed immediately following the brain MRI examination that already included post-contrast sequences, additional contrast administration was not pursued for the cervical spine study. TOF-MRA, which investigates blood flow noninvasively by suppressing stationary tissues and enhancing intravascular signals, was chosen as it is ideally suited to screen for vascular obstructions when conventional morphologic sequences were inconclusive (1, 2).

**Figure 2:**
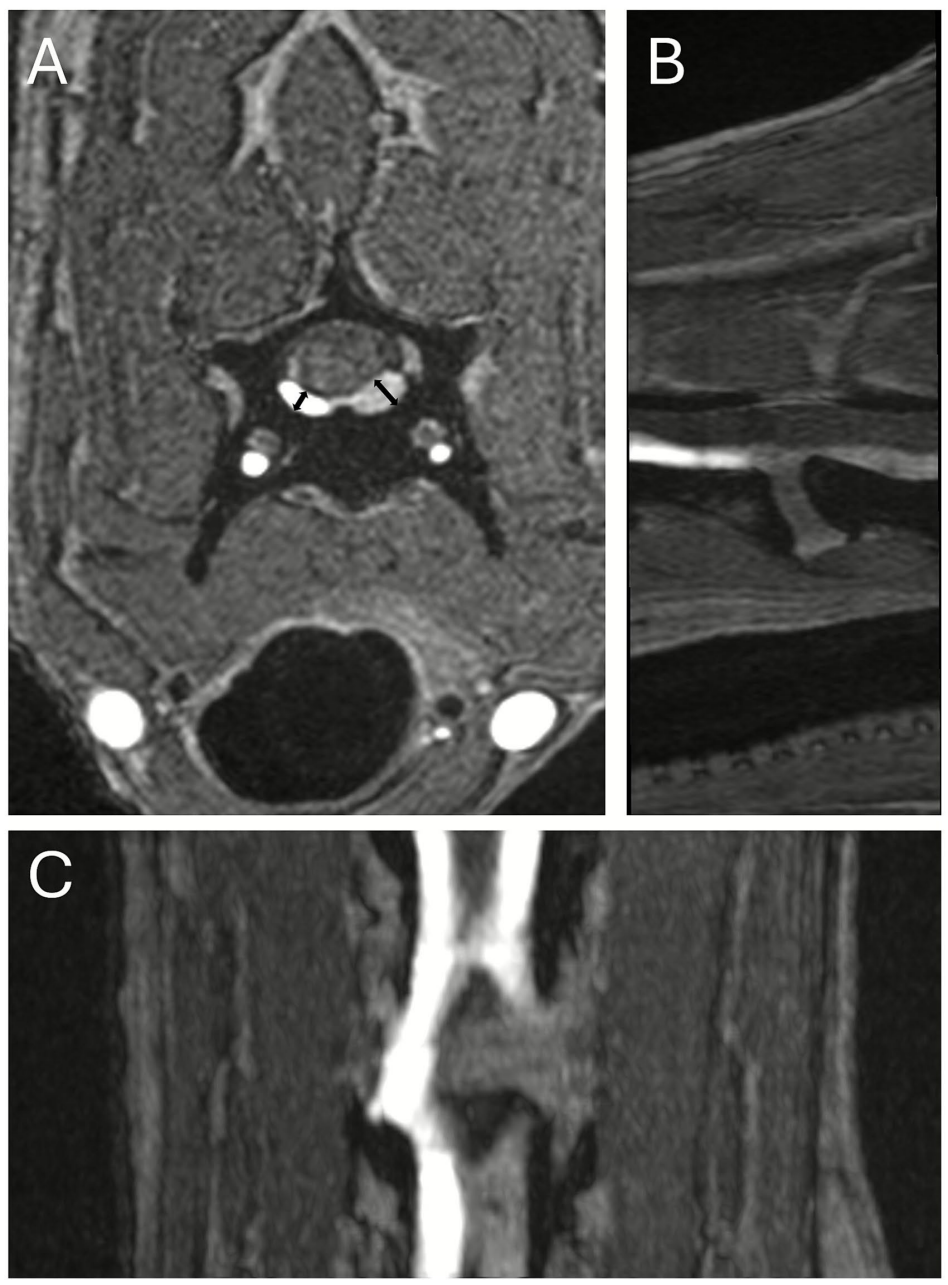
Multiplanar reconstruction of the TOF sequence in transverse **(A)**, parasagittal **(B)** and dorsal **(C)** planes at the level of C4-C5. A decreased signal intensity is observed in the left internal vertebral venous plexus (IVVP), extending from the caudal third of C4 to the caudal extent of the acquisition (caudal third of C5). The affected vessel on the left hand side shows an increased diameter compared with the contralateral IVVP (double-headed black arrows), measuring 2.1 mm in height on the right (patent) side and 3.3 mm on the left (thrombosed) side.

**Figure 3:**
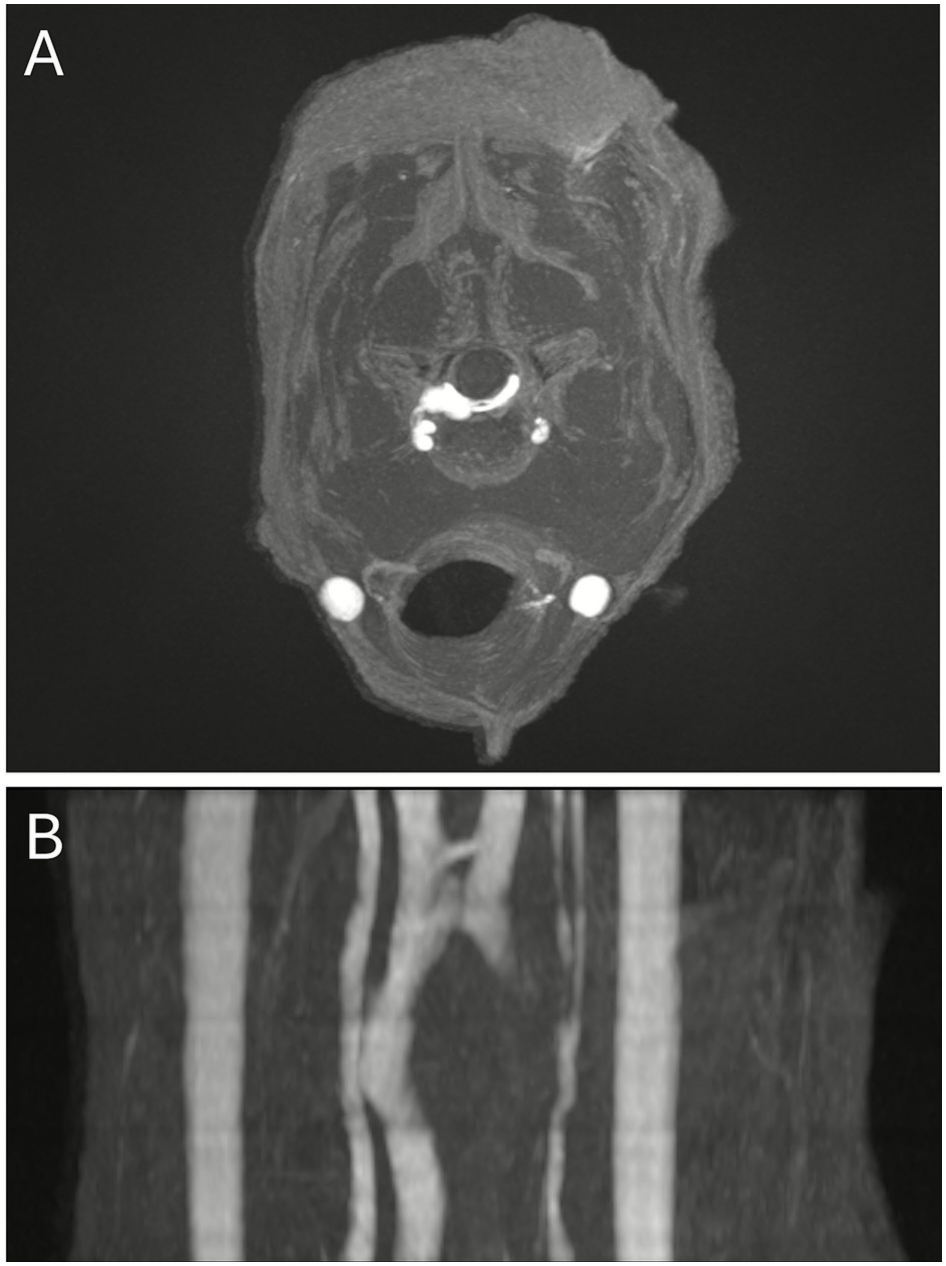
Maximum intensity projections of the TOF sequences. **(A)** Transverse image at the level of C4-C5 showing a decreased signal intensity of the left internal vertebral venous plexus. **(B)** Dorsal reconstruction confirming the unilateral distribution of the signal decrease within the left ventral internal vertebral venous plexus.

The TOF sequence revealed markedly decreased signal intensity in the left IVVP, extending from the caudal third of C4 vertebral body to the caudal extend of the acquisition (caudal third of C5), associated with an increase vessel diameter (up to 3.5 mm in height and 6.5 mm in width) compared to the contralateral vein (up to 2 mm in height and 5.5 mm in width) (Figures 2, 3).

A diagnosis of thrombosis of the left vein of the ventral branch of the IVVP extending from C4 to at least C5 was made, with unrelated multifocal intervertebral disc degeneration in the cervical region without spinal cord compression.

#### 2.2.3 Short term outcome

Patient was discharged the next day and prescribed rivaroxaban (0.5 mg/kg SID until 5 days before recheck), amoxicillin and clavulanic acid (13 mg/kg BID 5 days), gabapentin (5 mg/kg TID), levetiracetam (20 mg/kg TID) and ursodeoxycholic acid (8 mg/kg BID). In case of seizure activity, the patient was additionally prescribed midazolam (0.2 mg/kg) intranasally as SOS mediation. At recheck 1 month later the patient was asymptomatic and doing well. The owner reported no evidence of cervical pain and had stopped all medication prior to the recheck appointment.

## 3 Discussion

The decision to include a 3D TOF sequence in this patient was based on the acute onset of severe cervical pain, focal left thoracic limb lameness, generalized tonic–clonic epileptic seizures, and focal absent flow void in the left IVVP on conventional cervical MRI. The neurologic examination localized pain to the cervical spine, and evaluation of the T1- and T2-weighted images raised the suspicion of slow flow, thrombosis, or another form of vascular occlusion affecting the left IVVP.

The 3D TOF-MRA revealed an intraluminal flow void coupled with ipsilateral venous dilation—characteristic of an occlusive thrombus. Acute venous thrombi can appear as focal areas of absent flow signal within otherwise hyperintense venous channels on TOF, while subacute or chronic thrombi may show varying degrees of susceptibility effects or heterogeneous signal loss (9). The contiguous nature of the signal void in this dog’s IVVP, with sharply demarcated borders and absent flow-related enhancement, supports an embolic or *in situ* venous thrombosis, effectively ruling out flow artifacts or hypoplastic veins (8, 9).

The vertebral venous plexus is a valveless network permitting bidirectional flow; compression of one branch forces can redirect flow into collateral channels, resulting in turbulent flow and stasis (11). In the present case, mild degenerative changes at multiple cervical intervertebral discs were identified and although no clear compression was seen it is possible that the focal narrowing at C3/C4 could have contributed to an altered local venous flow pattern, promoting stasis in the left ventral branch of the IVVP.

The identification of IVVP thrombosis via TOF-MRA underscores TOF-MRA’s capacity for detecting vascular flow abnormalities (2) and provides additional support for its use in lesion localization, offering a diagnostic tool with possible therapeutic implications. TOF-MRA’s potential as a non-invasive tool for detecting spinal vascular lesions is particularly valuable for dogs with unexplained neurological signs, for example acute ataxia or pain, or those requiring serial imaging for chronic vascular conditions (10). Implementing TOF in standard spinal MRI protocols in such cases could facilitate early detection of venous thrombi or dural arteriovenous shunts and as such help guide appropriate therapeutic measures.

Concurrent laboratory abnormalities, including mild neutrophilia (10.83 × 10^9 cells/L; 2.6–8.9 × 10^9 cells/L) and elevated fibrinogen (404 mg/dL; reference 109–311 mg/dL) were found in this patient. The presence of systemic inflammation and an associated prothrombotic state were suspected. Systemic inflammation from a confirmed urinary tract infection (marked bacteriuria, leukocyturia, and proteinuria), possible chronic gastrointestinal issues, and advanced age may have predisposed this patient to epileptic seizures (12). Previous studies have shown that inflammatory cytokines (e.g., TNF-*α*, IL-6) released during infection can activate the coagulation cascade, promoting endothelial damage and thrombus formation (13). In our patient, although fibrinogen was elevated, other coagulation specific parameters (PT and PTT) as well as albumin were normal. While this may speak against a state of hypercoagulation, it has been shown that even without marked hypoalbuminemia, chronic enteropathy may lead to protein loss and a net reduction in anticoagulant factors (14, 15). In dogs with protein-losing enteropathies, hypercoagulability has been documented via thromboelastography—reflecting loss of antithrombin and other anticoagulant proteins (14, 15), unfortunately this was not carried out in this case. Advanced age, as was the case with this patient, can further predispose to thrombus formation, as aging is associated with endothelial dysfunction and increased procoagulant factors (16), however a prothrombotic state was not confirmed in this patient.

Given the absence of structural abnormalities on MRI of the brain and cervical spine, a direct causal relationship between the cervical venous thrombosis and the epileptic seizures appears unlikely. The seizures occurred as two isolated generalized tonic–clonic events, both in unfamiliar and potentially stressful environments (hospitalization ad the referring veterinarian and during abdominal ultrasound), while no seizures were observed at home. Notably, a severe cystitis was present at the time of both seizure episodes, and it cannot be excluded that stress and a concurrent systemic inflammatory state may have lowered the seizure threshold (12, 17). Nevertheless, an idiopathic epilepsy remains the main differential diagnosis for the epileptic seizures.

Limitations of TOF-MRA include susceptibility to motion artifacts and challenges in smaller breeds due to vessel caliber that may approach voxel dimensions resulting in poor signal-to-noise ratio and potential false negatives. An additional limitation in this case is the lack of histopathological confirmation of the suspected IVVP thrombosis, which was based on imaging findings and response to therapy.

Future prospective studies should evaluate TOF-MRA—both at 1.5 T and 3 T—in dogs with suspected venous thrombosis to establish sensitivity, specificity, and optimal imaging parameters. Correlation with postmortem findings or direct surgical observation will help validate imaging criteria. Quantitative flow-velocity measurements via phase-contrast imaging may further refine the distinction between slow flow and thrombus.

For patients with acute cervical pain without radiological evidence of spinal cord involvement, incorporating 3D TOF-MRA into cervical and thoracolumbar MRI protocol when routine sequences raise suspicion of altered vascular flow could be helpful for demonstrating vascular thrombosis. If a signal void in the vertebral venous plexus is identified on TOF-MRA, adjunctive hypercoagulability testing (e.g., thromboelastography, antithrombin activity) and timely initiation of anticoagulant therapy should be considered.

## Data Availability

The raw data supporting the conclusions of this article will be made available by the authors, without undue reservation.
